# The Evolving Landscape of Biomarkers for Anti-PD-1 or Anti-PD-L1 Therapy

**DOI:** 10.3390/jcm8101534

**Published:** 2019-09-25

**Authors:** Antje Tunger, Ulrich Sommer, Rebekka Wehner, Anne Sophie Kubasch, Marc-Oliver Grimm, Michael Philipp Bachmann, Uwe Platzbecker, Martin Bornhäuser, Gustavo Baretton, Marc Schmitz

**Affiliations:** 1 National Center for Tumor Diseases (NCT), University Hospital Carl Gustav Carus, TU Dresden, Fetscherstraße 74, 01307 Dresden, Germany; antje.tunger@uniklinikum-dresden.de (A.T.); rebekka.wehner@tu-dresden.de (R.W.); m.bachmann@hzdr.de (M.P.B.); martin.bornhaeuser@uniklinikum-dresden.de (M.B.); gustavo.baretton@uniklinikum-dresden.de (G.B.); 2 Institute of Immunology, Medical Faculty Carl Gustav Carus, TU Dresden, Fetscherstraße 74, 01307 Dresden, Germany; 3 Institute of Pathology, University Hospital Carl Gustav Carus, TU Dresden, Fetscherstraße 74, 01307 Dresden, Germany; ulrich.sommer2@uniklinikum-dresden.de; 4 Medical Clinic and Policlinic 1, Hematology and Cellular Therapy, Leipzig University Hospital, Liebigstraße 22, 04103 Leipzig, Germanyuwe.platzbecker@medizin.uni-leipzig.de (U.P.); 5 Department of Urology, Jena University Hospital, Lessingstraße 1, 07743 Jena, Germany; marc-oliver.grimm@med.uni-jena.de; 6 Department of Radioimmunology, Institute of Radiopharmaceutical Cancer Research, Helmholtz Center Dresden-Rossendorf, Bautzner Landstraße 400, 01328 Dresden, Germany; 7 German Cancer Consortium (DKTK), partner site Dresden, and German Cancer Centre (DKFZ), Im Neuenheimer Feld 280, 69120 Heidelberg, Germany; 8 Centre for Regenerative Therapies Dresden, TU Dresden, Fetscherstraße 105, 01307 Dresden, Germany; 9 Department of Medicine I, University Hospital Carl Gustav Carus, TU Dresden, Fetscherstraße 74, 01307 Dresden, Germany

**Keywords:** cancer immunotherapy, immune monitoring, immune checkpoints, programmed cell death protein 1, programmed cell death 1 ligand 1

## Abstract

The administration of antibodies blocking the immune checkpoint molecules programmed cell death protein 1 (PD-1) or programmed cell death 1 ligand 1 (PD-L1) has evolved as a very promising treatment option for cancer patients. PD-1/PD-L1 inhibition has significantly enhanced expansion, cytokine secretion, and cytotoxic activity of CD4^+^ and CD8^+^ T lymphocytes, resulting in enhanced antitumor responses. Anti-PD-1 or anti-PD-L1 therapy has induced tumor regression and improved clinical outcome in patients with different tumor entities, including melanoma, non-small-cell lung cancer, and renal cell carcinoma. These findings led to the approval of various anti-PD-1 or anti-PD-L1 antibodies for the treatment of tumor patients. However, the majority of patients have failed to respond to this treatment modality. Comprehensive immune monitoring of clinical trials led to the identification of potential biomarkers distinguishing between responders and non-responders, the discovery of modes of treatment resistance, and the design of improved immunotherapeutic strategies. In this review article, we summarize the evolving landscape of biomarkers for anti-PD-1 or anti-PD-L1 therapy.

## 1. Introduction

Recent studies revealed that targeting the immune checkpoint molecules programmed cell death protein 1 (PD-1) or programmed cell death 1 ligand 1 (PD-L1) by antibodies can significantly improve T cell-mediated antitumor responses and can induce objective clinical responses in about 20–40% of patients with different tumor types [1,2,3]. PD-1 is expressed on the cell surface of activated T cells, natural killer (NK) cells, and B cells, and binds to PD-L1 and PD-L2 [4,5]. PD-L1 is widely expressed on tumor cells, as well as hematopoietic and non-hematopoietic cells, and can be induced by proinflammatory cytokines such as interferon (IFN)-γ. PD-L2 has a higher affinity to PD-1, but its expression is more restricted, being mainly expressed by antigen-presenting cells (APCs) and induced mostly by interleukin-4 and granulocyte-macrophage colony-stimulating factor [6,7,8,9]. PD-L1 can also interact with CD80 on T cells, thereby delivering another inhibitory signal [10]. PD-1 plays an important role in modulating the immune response of antigen-experienced effector T cells. In peripheral tissues, activated T cells express PD-1 that binds to PD-L1 and PD-L2 on various tissue-associated cell types. PD-1 ligand expression is induced by proinflammatory molecules. PD-1 signaling results in inhibition of T cell proliferation, cytokine production, and cytotoxicity, protecting the tissue against collateral damage during immune responses [6,11,12]. This pathway is adopted by tumors to prevent themselves from immune attack [13].

Based on these findings, the efficacy of anti-PD-1 or anti-PD-L1 antibodies to significantly enhance T cell-mediated antitumor immunity has been explored in preclinical models. For example, it has been demonstrated in a murine pancreatic cancer model that anti-PD-1/PD-L1 blockade efficiently inhibits tumor growth [14]. When investigating the underlying mechanisms, an increased frequency of infiltrating CD8^+^ T cells as well as a higher expression of IFN-γ, granzyme B and perforin was found in tumors of treated mice. In another study, various patient-derived xenograft tumors were established in a humanized mouse model to explore the efficacy and mechanisms of anti-PD-1 therapy. Treatment with anti-PD-1 antibodies led to significant tumor growth inhibition, which was mediated by CD8^+^ T cells [15]. In clinical trials, anti-PD-1/PD-L1 blockade resulted in objective clinical responses in patients with melanoma [16,17,18], non-small-cell lung cancer (NSCLC) [19,20,21], renal cell carcinoma (RCC) [22,23], bladder cancer [24,25], and Hodgkin’s lymphoma [26]. More recently, it has been reported that overall survival (OS) curves show estimated 5-year rates of 34.2% among patients with melanoma, 27.7% among patients with RCC, and 15.6% among patients with NSCLC, indicating that anti-PD-1 treatment is associated with long-term survival in a subset of patients [27]. Due to their clinical efficacy, the U.S. Food and Drug Administration (FDA) approved anti-PD-1 or anti-PD-L1 antibody treatment for various tumor entities [28,29]. To further improve the clinical outcome in tumor patients, combinations of anti-PD-1/PD-L1 antibodies and other treatment options have been applied. For example, it has been reported that the combination of anti-PD-1 and anti-cytotoxic T lymphocyte antigen 4 (CTLA-4) antibody treatment results in a longer progression-free survival (PFS) than single agent therapy among previously untreated patients with metastatic melanoma [30]. In addition, objective response rates and OS were significantly higher with combined anti-PD-1 and anti-CTLA-4 therapy than with sunitinib among patients with previously untreated advanced RCC [31]. When combining anti-PD-1 treatment with chemotherapy, Paz-Ares et al. found that the addition of pembrolizumab to chemotherapy with carboplatin plus paclitaxel or nab-paclitaxel results in significantly longer OS and PFS than chemotherapy alone in patients with previously untreated metastatic, squamous NSCLC [32]. More recently, the clinical efficacy of anti-PD-1/PD-L1 antibodies and therapeutic strategies inhibiting the vascular endothelial growth factor (VEGF)/VEGF receptor axis has been evaluated in RCC patients. Thus, it has been reported that the combination of anti-PD-1 and axitinib leads to significantly longer OS and PFS compared to sunitinib among patients with previously untreated advanced RCC [33]. Furthermore, Rini et al. demonstrated that combined anti-PD-L1 and anti-VEGF therapy prolong PFS compared to sunitinib in RCC patients [34].

However, the majority of tumor patients do not respond to anti-PD-1/PD-L1 therapy. Therefore, the identification of biomarkers to predict treatment responders and therapy-related side effects is needed [35,36,37,38,39]. In addition, the discovery of mechanisms underlying anti-PD-1/PD-L1 therapy and modes of resistance is useful for improving current treatment modalities [35,36,37,38,39,40]. Here, we summarize recent findings on the PD-L1 expression or mutational load of tumor tissues, the frequency and phenotype of tumor-infiltrating or blood-circulating immune cells, and the microbiome in patients prior to and during anti-PD-1/PD-L1 treatment.

## 2. Results

### 2.1. Frequency and Phenotype of Tumor-Infiltrating Immune Cells

Previous reports provided evidence that the tumor immune contexture plays a critical role for the clinical outcome of patients [41,42,43]. For example, it has been shown that high densities of CD45RO^+^ T helper 1 cells and CD8^+^ T cells are associated with improved survival of colorectal cancer patients [44,45]. These results led to the development of a so-called “immunoscore” for an optimized tumor classification [46]. Accumulating evidence revealed that the tumor immune architecture also essentially contributes to the clinical efficacy of anti-PD-1/PD-L1 therapy. Thus, pretreatment melanoma samples from patients who displayed a clinical response to anti-PD-1 therapy showed higher CD8^+^ T cell frequencies compared to samples from patients with progressive disease during therapy [47], indicating that pre-existing tumor-infiltrating CD8^+^ T cells are predictors of a clinical response to PD-1 blockade. Another study revealed that a high T effector gene signature expression in tumor tissues at baseline was associated with improved overall response rate and PFS of RCC patients treated with anti-PD-L1 and anti-VEGF antibodies [48]. In contrast, a high myeloid inflammation gene signature expression was correlated with reduced PFS in patients receiving anti-PD-L1 alone or anti-PD-L1 and VEGF antibodies. Moreover, Thommen et al. identified three distinct intratumoral CD8^+^ T cell subsets based on PD-1 expression in NSCLC patients [49]. Besides the CD8^+^ T cell subsets with intermediate (PD-1^N^) and no PD-1 expression, a subpopulation with high PD-1 expression (PD-1^T^) was detected that showed a markedly different transcriptional and metabolic profile. The presence of PD-1^T^ T cells was strongly predictive for the clinical outcome of anti-PD-1-treated NSCLC patients [49]. Single cell profiling of tumor-infiltrating immune cells from 32 metastatic melanoma patients treated with anti-PD-1- and/or CTLA-4 antibodies identified two major CD8^+^ T cell states associated with clinical outcome [50]. CD8^+^ T cells with increased expression of genes linked to memory, activation and cell survival were enriched in responding melanoma lesions. In contrast, CD8^+^ T cells with increased expression of genes linked to exhaustion were enriched in non-responding lesions. Thus, the ratio of memory-like to exhausted CD8^+^ T cells was associated with response to checkpoint inhibitor (CPI) therapy. In addition, elevated frequencies of melanoma-infiltrating TCF7^+^CD8^+^ T cells were found to predict positive clinical outcome in an independent cohort of anti-PD-1-treated patients [50].

Further studies explored the impact of anti-PD-1 blockade on tumor-infiltrating T cells (TILs). Thus, it has been shown that PD-1 therapy preferentially induces proliferation of exhausted-like tumor-infiltrating CD8^+^ T lymphocytes [51]. Furthermore, Ribas et al. reported that anti-PD-1 blockade leads to a higher TIL density in treatment responders [52]. In addition, it has been found that CD4^+^FoxP3^-^PD-1^hi^ T cells (4PD-1^hi^) accumulate intratumorally in NSCLC patients and that a lack of effective 4PD-1^hi^ reduction after anti-PD-1 therapy correlates with poor prognosis [53]. Taube et al. investigated the infiltrating immune cells of PD-L1^+^ and PD-L1^−^ melanomas and found an IFN-γ-dominated cytokine expression pattern in TILs of PD-L1^+^ melanomas [13]. This can be explained by the mechanism that the recognition of tumor antigens by activated TILs leads to a PD-1 upregulation and induction of IFN-γ secretion. Consequently, tumor cells enhance PD-L1 expression, thereby protecting the tumor from PD-1^+^ effector T cell-mediated elimination.

Verma et al. demonstrated that the status of CD8^+^ T cell priming essentially contributes to anti-PD-1 therapeutic resistance [54]. Thus, PD-1 blockade in unprimed or suboptimally primed CD8^+^ T cell conditions resulted in the induction of dysfunctional PD-1^+^CD38^hi^ CD8^+^ cells, leading to anti-PD-1 antibody resistance and treatment failure. In contrast, PD-1 blockade of optimally primed CD8^+^ T lymphocytes prevented the induction of dysfunctional CD8^+^ cells, reversing resistance. They also reported that a high frequency of tumor-infiltrating or blood-circulating PD-1^+^CD38^hi^ CD8^+^ cells in melanoma patients can serve as a biomarker of anti-PD-1 resistance.

### 2.2. PD-L1 Expression by Tumor Cells and Tumor-Infiltrating Immune Cells

In order to treat a patient with an antibody directed to PD-1 or PD-L1, a positive PD-L1 status is required for the majority of tumor entities, at least in first-line therapy. This currently applies to NSCLC, gastric or gastroesophageal junction adenocarcinoma, cervical cancer, urothelial carcinoma, head and neck squamous cell carcinoma, and esophageal squamous cell carcinoma [55]. In this case, testing of the tissue before therapy is mandatory in the sense of a so-called “companion diagnostic”, whereby the situation in Europe (testing is required, open methods) differs from the situation in the sphere of influence of the FDA (testing with a specific diagnostic antibody required).

Various clinical studies have shown that patients with a higher PD-L1 expression had a better response to a corresponding therapy [56,57,58]. Unlike, for example, a genetic test, PD-L1 expression is not a “digital” marker in the sense of present/not present, but a value that must be defined as positive or negative according to empirical surveys. The current IMpassion 130 study on triple negative breast carcinomas showed that even very low positivity rates of 1% can be sufficient. This study compared PD-L1 blockade and nab-paclitaxel versus placebo and nab-paclitaxel in metastatic tumors that had not previously undergone systemic therapy. An expression on ≥ 1% of intra- and peritumoral immune cells was defined as PD-L1 positive, which was the case in 40.9% of the total 902 patients. A consistent benefit was observed for PFS and OS across the PD-L1 positive subgroup, whereas no efficacy benefit was seen in the PD-L1 negative subgroup. Interestingly, however, this does not seem to be a linear relationship, in the sense of a continuously improved response at expression rates higher than 1% [59]. The fact that a positive PD-L1 status not only serves to optimize response rates for successful therapy, but can also protect patients from damage, was recently described in urothelial carcinoma of the urinary bladder. In first-line therapy with anti-PD-L1 antibodies (IMvigor 130) and anti-PD-1 antibodies (Keynote-361), an increased mortality rate [60,61,62] was observed in the group of PD-L1 negative patients treated with CPIs. However, the immunohistochemically-determined PD-L1 status as a biomarker can only be an approximation, which is also shown by the fact that patients do not respond clinically to an appropriate therapy despite a positive PD-L1 status. Thus, it has been demonstrated that RCC patients with 1% or greater PD-L1 expression have reduced OS compared to patients with less than 1% [23]. These patients were previously treated with antiangiogenic agents and then assigned to receive anti-PD-1 treatment. Furthermore, Gettinger et al. found no clear association between PD-L1 expression and survival in NSCLC patients receiving anti-PD-1 therapy [63].

Although the immunohistochemically-determined PD-L1 status is not an optimal biomarker to predict a response to CPI therapy, it is currently the best established and validated marker. The fact that the test is fast, material- and cost-saving and can be carried out in almost any pathological institute is also a clear advantage of this method. One of the decisive challenges here is not so much the technical implementation of the staining as the correct interpretation and evaluation, which is also reflected by the results of the interlaboratory comparisons within the framework of quality assurance in pathology [64]. For pathologists, the complexity lies in the multitude of available antibodies or assays, each of which has its own scoring algorithms and cut-off values, which can differ from entity to entity, and also the question of whether tumor and/or immune cells should be tested. The PD-L1 status can be specified using various scores. IC scoring (e.g., for first-line therapy with atezolizumab in urothelial carcinoma) only considers immune cells, not expression on tumor cells. The expression is evaluated as a percentage of the tumor area in four categories (IC 0 to IC 3; <1% to ≥10%) [65]. Another approach underlies the dimensionless “combined positivity score—CPS”. In this evaluation strategy, both tumor and immune cells are taken into account, whereby the expression of tumor and immune cells is jointly related to the population of tumor cells. This means that a value greater than 100 is possible here; however, it is limited to the maximum value of 100 [66]. Another score applied in practice is the “tumor proportion score—TPS”, which reports the percentage of immunohistochemically positive, epithelial tumor cells in the entire tumor. In general, it has been shown that the various scoring algorithms can be implemented accurately and with high reproducibility by pathologists after appropriate training [67,68,69].

On questions of analytical comparability and technical reproducibility of immunohistochemical staining, great efforts have been made both at national and international level, which essentially show that the assays and antibodies 22C3, 28-8 and SP263 used in clinical studies can be regarded as comparable and interchangeable with regard to immune cells. A similar picture can be seen with tumor cells, where SP142 has a reduced sensitivity and therefore detects fewer tumor cells than the other assays mentioned above [68,69,70,71,72]. This difference in staining and detection behavior should be considered, especially if not only the IC score but also the CPS has to be determined on a sample.

### 2.3. Tumor Mutational and Neoantigen Burden

Accumulating evidence suggests that the tumor mutational burden (TMB) can significantly influence the immune contexture and the sensitivity to CPI therapy. A higher mutational load increases the probability of immunogenic mutations, providing neoantigens for T cell responses. Recent studies explored the impact of the TMB and derived neoantigens on the sensitivity of tumors to PD-1 blockade. Thus, it has been reported that a higher non-synonymous mutation or candidate neoantigen burden in tumors was associated with improved PFS of anti-PD-1-treated NSCLC patients [73]. In addition, Le et al. demonstrated that anti-PD-1 antibody-treated patients with mismatch repair-deficient colorectal cancer showed a higher immune-related objective response rate and immune-related PFS rate than patients with mismatch repair-proficient colorectal cancer [74]. Analyzing the sequence of the whole exome revealed that tumors with a deficiency in mismatch repair displayed a significantly higher number of somatic mutations per tumor compared to tumors proficient in mismatch repair. Longer PFS was correlated with high numbers of somatic mutations and potential mutation-associated neoantigens [74]. More recently, they extended the study to explore the clinical outcome of anti-PD-1-treated patients with advanced mismatch repair-deficient cancers across 12 different tumor types [75]. Objective radiographic responses were observed in 53% of patients, and complete responses were achieved in 21% of patients, indicating that a mismatch repair-deficiency can make the tumor sensitive to PD-1 blockade, regardless of the cancers’ tissue of origin. Another study found 70% objective clinical responses, including 32% complete responses in anti-PD-1-treated patients with desmoplastic melanoma [76]. The high number of detected somatic mutations and a frequent pre-existing adaptive immune response may essentially contribute to the impressive clinical response rates. Furthermore, it has been reported that anti-PD-1 antibody-treated RCC patients bearing loss-of-function mutations in the *PBRM1* gene experienced a clinical benefit [77]. These findings were confirmed in an independent validation cohort of 63 patients treated with PD-1 or PD-L1 blockade therapy alone or in combination with anti-CTLA-4 [77]. In contrast, McDermott et al. found no correlation between mutational or neoantigen burden and PFS of ccRCC patients treated with anti-PD-L1 alone or combined with anti-VEGF antibodies [48]. Notably, mutations in tumors can also be associated with resistance to PD-1 blockade. Thus, it has been reported that the anti-PD-1-treated subgroup of KRAS-mutant lung adenocarcinoma patients with a *STK11*/*LKB1* co-mutation exhibits shorter PFS and OS compared to the only KRAS-mutant lung adenocarcinoma cohort or the KRAS- and TP53-mutant lung adenocarcinoma subgroup. These findings provide evidence that *STK11*/*LKB1* may be critical for anti-PD-1 resistance in KRAS-mutant lung adenocarcinoma patients [78].

When investigating an association between intratumoral neoantigen load and responsiveness to PD-1 blockade, McGranahan et al. found that a high clonal neoantigen burden in tumors of anti-PD-1 antibody-treated NSCLC patients is associated with improved clinical outcome [79]. In addition, Łuksza et al. described a neoantigen fitness model based on the likelihood of neoantigen presentation by HLA molecules and subsequent T cell recognition, which is able to predict clinical outcome of anti-PD-1 antibody-treated tumor patients [80]. Riaz et al. investigated the mechanisms by which PD-1 blockade modulates tumor evolution during therapy. Therefore, genomic changes in tumors from 68 patients with advanced melanomas, who progressed on anti-CTLA-4 treatment or were anti-CTLA-4 therapy-naïve, before and after anti-PD-1 initiation were determined [81]. After 4 weeks of anti-PD-1 therapy, a marked reduction of mutation and neoantigen load among patients with objective clinical responses was observed.

Chowell et al. explored the impact of the HLA class I genotype on the response of a large cohort of advanced cancer patients treated with anti-CTLA-4 and/or anti-PD-1 antibodies [82]. Maximal heterozygosity at HLA class I loci improved OS after CPI therapy compared with patients who were homozygous for at least one HLA class I locus, indicating that the HLA class I genotype significantly influences tumor sensitivity to this treatment modality.

Further studies discovered mechanisms of resistance to PD-1 blockade therapy in tumor patients. Thus, loss-of-function mutations in genes encoding interferon-receptor-associated Janus kinase 1 (*JAK1*) or *JAK2* were detected, resulting in a lack of response to IFN-γ-mediated antitumor effects [83]. Another mode of resistance is based on a defective antigen presentation by tumor cells. Point mutations, deletions or loss of heterozygosity in beta-2-microglobulin (*B2M*) were found, leading to an insufficient HLA class I surface expression [83,84,85].

### 2.4. Frequency and Phenotype of Blood-Circulating Immune Cells and Soluble Molecules

Further studies investigated the frequency and phenotype of circulating immune cells and the concentration of soluble molecules in blood from anti-PD-1-treated tumor patients to identify potential biomarkers for the prediction of clinical responses. Thus, anti-PD-1 treatment resulted in an expansion of blood-circulating PD-1^+^ CD8^+^ T cells in NSCLC patients [86]. PD-1^+^ CD8^+^ T cell responses were observed in the majority of patients with clinical benefit. In addition, Takeuchi et al. found an increase in a subset of central memory CD4^+^ T cells, defined by the phenotype CD27^+^Fas^-^CD45RA^-^CCR7^+^, in blood of melanoma patients after anti-PD-1 treatment, which is associated with clinical responses [87]. A further study revealed that the magnitude of reinvigoration of T cells with an exhausted phenotype determined in relation to pretreatment tumor burden is correlated with clinical responses in anti-PD-1-treated melanoma patients [88]. More recently, it has been shown that PD-1 expression was significantly higher on CD4^+^ T cells and T-cell immunoglobulin and mucin containing protein 3 (TIM-3) expression was significantly higher on CD8^+^ T cells in blood of melanoma patients that respond to anti-PD-1 therapy compared to non-responders [89]. However, it is not only the T cell compartment that may serve as a potential biomarker for clinical responses. Krieg et al. reported that the frequency of classical blood monocytes at baseline in anti-PD-1-treated melanoma patients is a predictor of PFS and OS [90]. Furthermore, high relative eosinophil counts and relative lymphocyte counts in peripheral blood at baseline were associated with favorable OS of melanoma patients receiving anti-PD-1 antibodies [91].

When exploring soluble molecules as potential biomarkers for treatment response, it has been demonstrated that low lactate dehydrogenase (LDH) in blood at baseline was correlated with improved OS in anti-PD-1-treated melanoma patients [91]. Significantly lower baseline serum levels of monocyte chemoattractant protein 1, leucocyte inhibition factor, and CTLA-4 were associated with hyperprogressive metastatic gastrointestinal cancer among patients receiving CPI therapy [92]. Chow et al. reported that intratumoral expression and activity of the chemokine receptor CXCR3 is crucial for the efficacy of anti-PD-1 treatment in tumor-bearing mice [93]. In this context, CXCR3 was required for improving the intratumoral CD8^+^ T cell response, but not for the CD8^+^ T cell migration into the tumor. When investigating the plasma concentration of the CXCR3 ligands CXCL9 and CXCL10 in tumor patients treated with anti-PD-1 alone or anti-PD-1 and anti-CTLA-4 antibodies, it was found that the treatment responder group has elevated levels within the first few months after therapy. Moreover, Chen et al. observed an increase of melanoma-derived exosomes carrying PD-L1 on their surface in the blood of patients during early stages of anti-PD-1 therapy [94]. This phenomenon is associated with clinical responses, indicating that exosomal PD-L1 can serve as a predictor for anti-PD-1 therapy.

As described above, several studies have shown that a high TMB in anti-PD-1 antibody-treated patients is associated with improved clinical outcome. Following this finding, Gandara et al. explored the potential of TMB in blood of anti-PD-L1-treated NSCLC patients as a predictor of clinical benefit [95]. They found that blood-based TMB identifies patients with significant improvements in PFS.

### 2.5. Microbiome

Recent findings in tumor patients indicate that the diversity and composition of the gut microbiota have an impact on the efficacy of CPI therapy [96]. Thus, a correlation between the gut microbiome of tumor patients and their clinical response to anti-PD-1 blockade has been discovered [97,98]. The gut microbiome of responding melanoma patients showed a significantly higher alpha diversity and a relative abundance of bacteria of the *Ruminococcaceae* family [97]. The *Bacteroidales* order was enriched in non-responders. Immune profiling revealed a higher density of melanoma-infiltrating CD8^+^ T cells at baseline and a higher frequency of blood-circulating CD8^+^ and CD4^+^ T cells in responder patients with a favorable gut microbiome [97]. In another study, the bacterial species *Bifidobacterium longum*, *Collinsella aerofaciens*, and *Enterococcus faecium* were more abundant in responders [99]. Routy and colleagues found that the relative abundance of *Akkermansia munciniphila* is significantly associated with a favorable clinical outcome of patients with advanced NSCLC, RCC, and urothelial carcinoma [98]. These findings indicate that the efficacy of anti-PD-1 therapy can be markedly influenced by the gut microbiome in tumor patients. Based on these findings, the impact of antibiotic use on the efficacy of PD-1 blockade has been investigated. Notably, PFS and OS were significantly shorter in antibiotic-treated patients [98]. In line with this study, it has been reported that antibiotic use within 30 days prior to the initiation of CPI treatment was associated with decreased PFS [100]. These results provide evidence that antibiotics may significantly influence the efficacy of CPI-based therapy in tumor patients.

## 3. Conclusions

Targeting the PD-1/PD-L1 axis can induce objective clinical responses and improve survival in patients with various tumor types such as melanoma, NSCLC, and RCC. However, the majority of tumor patients do not respond to anti-PD-1/PD-L1 therapy. Various potential biomarkers in the blood or tumor tissues of anti-PD-1/PD-L1-treated patients have been discovered that may predict clinical outcome and, therefore, may discriminate between treatment responders and non-responders. However, clinical trials have yielded contradictory results concerning the suitability of some of these potential biomarkers. In addition, the potential of some newly described biomarkers has to be validated in large cohorts of anti-PD-1/PD-L1-treated patients. Based on these issues, a single biomarker that can be widely used to predict the clinical outcome is not available. The design of predictive models combining the most promising single biomarkers described so far may improve the identification of patients who will benefit from anti-PD-1/PD-L1 therapy.

Recently, various mechanisms of resistance underlying an inefficient anti-PD-1/PD-L1 therapy have been discovered. Based on these findings, improved treatment modalities can be designed. Thus, a low degree of tumor-infiltrating T cells before anti-PD-1/PD-L1 therapy is associated with progressive disease during treatment. This suggests that the administration of drugs that promote T cell trafficking to tumors before anti-PD-1/PD-L1 therapy may improve the clinical outcome for patients with an insufficient density of pre-existing tumor-infiltrating T cells. This may be achieved by the application of epigenetic modulators that remove the repressed intratumoral secretion of T cell-attracting chemokines, leading to increased T cell infiltration. Furthermore, PD-1 blockade in unprimed or suboptimally primed CD8^+^ T cell conditions induces dysfunctional CD8^+^ T cells, resulting in anti-PD-1 antibody resistance. This observation indicates that the generation of optimally primed CD8^+^ T lymphocytes by activating strategies such as adjuvant-based vaccination or radiotherapy may prevent resistance to anti-PD-1 therapy.

## Summary

**Figure 1:**
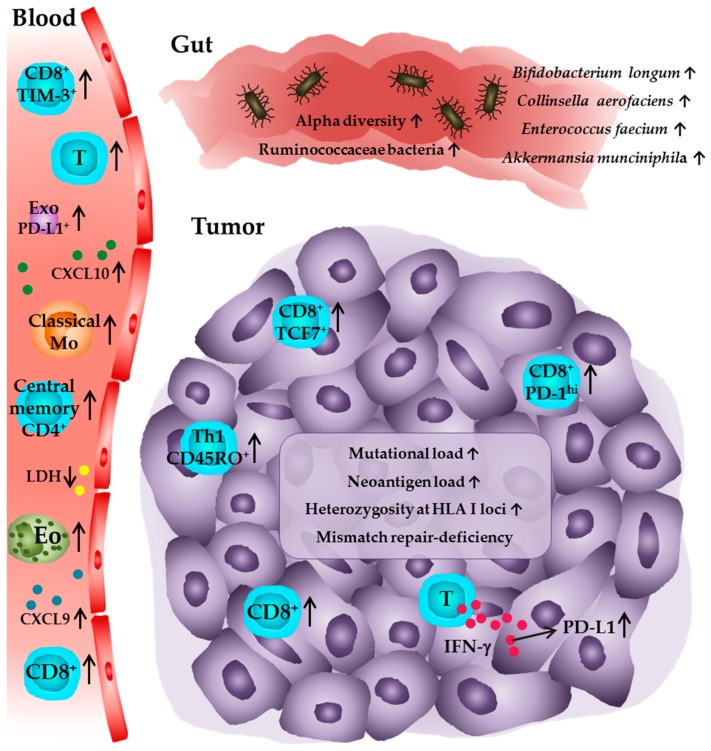
Immune profile of tumor patients receiving anti-PD-1 or anti-PD-L1 antibodies associated with favorable clinical outcome. In peripheral blood, higher numbers of eosinophils, lymphocytes, PD-1^+^ T cells, TIM-3^+^ CD8^+^ T cells, central memory CD4^+^ T cells, and classical monocytes are associated with improved clinical responses. Furthermore, low levels of LDH and elevated levels of CXCL9, CXCL10, as well as PD-L1^+^ exosomes are predictive for a clinical outcome. Within the tumor, higher densities of CD8^+^ T cells in pretreatment tumor samples and an increase in intratumoral CD8^+^ T cell frequencies during anti-PD-1 therapy are detectable in patients that show a clinical response. The presence of PD-1^T^ T cells and an elevated frequency of TCF7^+^CD8^+^ T cells is also associated with a favorable outcome as well as high numbers of CD45RO^+^ Th1 cells and high PD-L1 expression on tumor cells and infiltrating immune cells. Further studies indicate that a high intratumoral mutational and neoantigen load, heterozygosity at the HLA I loci, and a deficiency in the mismatch repair system, which may lead to a higher level of somatic mutations in the tumor cells, are correlated with an improved survival of patients. In addition, a significantly higher alpha diversity or a relative abundance of Ruminococcaceae bacteria as well as of the bacterial species *Akkermansia munciniphila*, *Bifidobacterium longum*, *Collinsella aerofaciens*, and *Enterococcus faecium* in the gut microbiome are associated with a favorable clinical outcome for tumor patients. Eo—eosinophil; T—T cell; Mo—monocyte; Exo—exosome; Th1—T helper cell type.

A summary of potential biomarkers for the prediction of clinical responses to anti-PD-1/PD-L1 therapy is given in Figure 1 and Table 1.

## Figures and Tables

**Table 1:** Biomarkers associated with clinical response to anti-PD-1 or anti-PD-L1 treatment.

Biomarker	Association with Clinical Outcome	Malignancy	Treatment	Tissue Type for Biomarker Assessment	References
Pre-existing and highly frequent tumor-infiltrating CD8^+^ T cells	Positive	Melanoma	Anti-PD-1	Tumor tissue	[47,52]
High T effector gene signature expression	Positive	RCC	Anti-PD-L1 and anti-VEGF	Tumor tissue	[48]
High myeloid inflammation gene signature expression	Negative	RCC	Anti-PD-L1/Anti-PD-L1 and anti-VEGF	Tumor tissue	[48]
PD-1^hi^CD8^+^ T cells	Positive	NSCLC	Anti-PD-1	Tumor tissue	[49]
Ratio memory-like to exhausted CD8^+^ T cells	Positive	Melanoma	Anti-PD-1 and/or anti-CTLA-4	Tumor tissue	[50]
TCF7^+^CD8^+^ T cells	Positive	Melanoma	Anti-PD-1	Tumor tissue	[50]
CD4^+^FoxP3^-^PD-1^hi^ T cells	Negative	NSCLC	Anti-PD-1	Tumor tissue	[53]
PD-1^+^CD38^hi^CD8^+^ T cells	Negative	Melanoma	Anti-PD-1	Tumor tissue, blood	[54]
PD-L1 expression	Positive	Multiple cancer types	Anti-PD-1/Anti-PD-L1	Tumor tissue	[56,57,58]
Tumor mutational and neoantigen load	Positive	NSCLC	Anti-PD-1/Anti-PD-L1	Tumor tissue, blood	[73,79,95]
Mismatch repair-deficiency	Positive	Multiple cancer types	Anti-PD-1	Tumor tissue	[74,75,76]
Loss-of-function mutations in *PBRM1*	Positive	RCC	Anti-PD-1/Anti-PD-L/Anti-PD-1 or anti-PD-L1 and anti-CTLA-4	Tumor tissue	[77]
*STK11/LKB1* mutations	Negative	KRAS-mutant lung adeno-carcinoma	Anti-PD-1	Tumor tissue	[78]
Reduction of mutational and neoantigen load under therapy	Positive	Melanoma	Anti-PD-1	Tumor tissue	[81]
HLA class I heterozygosity	Positive	Melanoma, NSCLC	Anti-PD-1 and/or anti-CTLA-4	Blood	[82]
*JAK1*/*JAK2* mutations	Negative	Melanoma	Anti-PD-1	Tumor tissue	[83]
*B2M* mutations	Negative	Melanoma, NSCLC	Anti-PD-1	Tumor tissue	[83,84,85]
PD-1^+^CD8^+^ T cells	Positive	NSCLC	Anti-PD-1	Blood	[86]
CD27^+^Fas^-^CD45RA^-^CCR7^+^CD4^+^ T cells	Positive	Melanoma	Anti-PD-1	Blood	[87]
PD-1^+^CD4^+^ T cells	Positive	Melanoma	Anti-PD-1	Blood	[89]
TIM-3^+^CD8^+^ T cells	Positive	Melanoma	Anti-PD-1	Blood	[89]
Classical monocytes	Positive	Melanoma	Anti-PD-1	Blood	[90]
High relative eosinophil counts	Positive	Melanoma	Anti-PD-1	Blood	[91]
High relative lymphocyte counts	Positive	Melanoma	Anti-PD-1	Blood	[91]
Low LDH	Positive	Melanoma	Anti-PD-1	Blood	[91]
Low MCP1, LIF, CTLA-4	Negative	Gastro-intestinal cancer	Anti-PD-1/Anti-PD-L1/Anti-CTLA-4	Blood	[92]
High CXCL9 and CXCL10	Positive	Melanoma	Anti-PD-1/Anti-PD-1 and anti-CTLA-4	Blood	[93]
PD-L1^+^ Exosomes	Positive	Melanoma	Anti-PD-1	Blood	[94]
High alpha diversity	Positive	Melanoma	Anti-PD-1	Gut	[97]
Bacteria of the *Ruminococcaceae* family	Positive	Melanoma	Anti-PD-1	Gut	[97]
Bacteria of the *Bacteroidales* order	Negative	Melanoma	Anti-PD-1	Gut	[97]
*Bifidobacterium longum*, *Collinsella aerofaciens*, *Enterococcus faecium*	Positive	Melanoma	Anti-PD-1	Gut	[99]
*Akkermansia munciniphila*	Positive	NSCLC, RCC, urothelial carcinoma	Anti-PD-1	Gut	[98]
